# Developmental Changes in Locating Voice and Sound in Space

**DOI:** 10.3389/fpsyg.2017.01574

**Published:** 2017-09-20

**Authors:** Emiko Kezuka, Sachiko Amano, Vasudevi Reddy

**Affiliations:** ^1^Faculty of Literature, Gunma Prefectural Women’s University Gunma, Japan; ^2^Faculty of Nutrition, Kagawa Nutrition University Saitama, Japan; ^3^Department of Psychology, University of Portsmouth Portsmouth, United Kingdom

**Keywords:** infants, localization, voice, sound, multi-modal space, perseverative errors, triadic interactions

## Abstract

We know little about how infants locate voice and sound in a complex multi-modal space. Using a naturalistic laboratory experiment the present study tested 35 infants at 3 ages: 4 months (15 infants), 5 months (12 infants), and 7 months (8 infants). While they were engaged frontally with one experimenter, infants were presented with (a) a second experimenter’s voice and (b) castanet sounds from three different locations (left, right, and behind). There were clear increases with age in the successful localization of sounds from all directions, and a decrease in the number of repetitions required for success. Nonetheless even at 4 months two-thirds of the infants attempted to search for the voice or sound. At all ages localizing sounds from behind was more difficult and was clearly present only at 7 months. Perseverative errors (looking at the last location) were present at all ages and appeared to be task specific (only present in the 7 month-olds for the behind location). Spontaneous attention shifts by the infants between the two experimenters, evident at 7 months, suggest early evidence for infant initiation of triadic attentional engagements. There was no advantage found for voice over castanet sounds in this study. Auditory localization is a complex and contextual process emerging gradually in the first half of the first year.

## Introduction

The present study uses a semi-naturalistic experimental setting to study infants’ tendencies to search for and locate a voice and a sound in different locations around them while face to face with another person. The ability to locate sounds in space by turning toward them is present even in neonates (Wertheimer, 1961; Butterworth and Castillo, 1976; Alegria and Noirot, 1978; Muir and Field, 1979; Castillo and Butterworth, 1981), suggesting a very early coordination of auditory and visual space. That this ability may be crucial for typical development is suggested by findings of a suboptimal integration of information and differential localizing of sounds in autism (Dawson et al., 2004; Nadig et al., 2007; Skewes and Gebauer, 2016). However, the early development of this ability is not well-understood, particularly in complex multi-modal spaces as in typical everyday environments.

Although the ability continues to develop through infancy (Ashmead et al., 1987; Morrongiello and Rocca, 1987; Morrongiello, 1988; Morrongiello et al., 1990; Clifton et al., 1991) there is a suggestion that its development is not linear. One study found a U-shaped curve in early localization of a rattle sound, with a dip in performance between 1 and 3 months and then a rise again at 4–5 months (Muir et al., 1989). The reasons for this U-shaped trajectory may involve many other factors in addition to a possible maturational shift from sub-cortical to cortical control as suggested by the authors. Factors such as the opportunity to practice, for instance, are known to modulate phenomena such as primary walking previously believed to be reflective of sub-cortical control shifting later to cortical control as secondary walking (Thomas and Saint-Anne Dargassies, 1952; Thelen et al., 1991).

Turning the head to look for a sound is not always a simple orienting response but may also be an expectation of something worth seeing. As such, it may well be the case that attempting to locate the voice of a person has a different interest to infants than the sound of an object. There is a very early preference in humans for turning toward, looking longer at and following, human face-like visual stimuli (Goren et al., 1975; Johnson et al., 1991), mother’s voices (DeCasper and Fifer, 1980), sounds in the human voice range (Shultz and Vouloumanos, 2010; Vouloumanos et al., 2010; Vouloumanos and Waxman, 2014) and human milk smells (Macfarlane, 1975). In the study of other cognitive skills involving locating objects or persons – such as the drop in perseveration errors in object permanence – some studies have found that person permanence is ‘easier’ and is apparent earlier than, object permanence (Bell, 1970; Bowlby, 1980/1998). However, other studies have found that if the conditions are strictly comparable the person over object advantage disappears (Jackson et al., 1978). It is not clear whether the developmental trajectory suggested for localization in the early months applies equally to both voices and sounds.

We do not yet know the extent to which the presence of multiple persons and objects in a typical environment might influence the infant’s ability to locate sounds or about the social expectations involved in localization. In their ‘split mother’ experiment, Aronson and Rosenbloom (1971) focused on infants’ daily life space, and reported that infants as young as 28 days (age range from 28 to 56 days) became distressed upon observing their mothers speak to them while their voice was displaced in space (achieved through controlling a stereo system with two speakers). They said that the spatial dislocation was a violation of the young infants’ perceptual world, in which the speaker and the voice typically share the same spatial location. However, several studies report failure to replicate or confirm the finding that infants were distressed by discrepancies, in infants of about the same age (39–58 days old, Condry et al., 1977) and in an older age group (1, 4, and 7 months of age, McGurk and Lewis, 1974). McGurk and Lewis, however, observed that infants at 4 and 7 months responded to the mother’s voice from an active loudspeaker, indicating that older infants’ sound localization was active, even while they continued looking at their mothers’ faces, but these infants did not show distress. Their results suggest that 4- to 7-month-olds seem to have a relatively high tolerance for audio-visual spatial discrepancy. No following replication of the Aronson and Rosenbloom experiment has been reported since then, and it remains unclear whether young infants are indeed efficient at sound localization, or are tolerant of audio-visual spatial discrepancy.

Critically, it remains unclear how localization functions in a multi-person environment. While infants even at 12 weeks (Fivaz-Depeursinge et al., 2005) may have no difficulty in switching attention from one parent to another in close proximity, it is not clear how this would work in terms of turning to search for a voice when the other adult is not immediately visible. We do not yet know when or how infants in real-world situations begin to search for and localize voices and other social sounds coming from different locations in space. The ‘split mother’ situation may be too far from the real world, but there may be many other situations in daily life where infants are called by someone from outside of view while they are talking to another person in front of them. When different visual figures, voices and sounds are coming from all angles together, it is likely to be particularly important for infants to be able to locate in space and identify someone familiar and reliable among the many voices and figures. One may also see, in real-life multi-person situations (Fivaz-Depeursinge et al., 2010) simple precursors to triadic engagements such as spontaneous shifting of attention from one adult to the other.

One crucial aspect of the space within which localization needs to occur is whether the ‘object’ to be located is at least potentially peripherally visible to the infant or whether it is in ‘invisible’ space, behind the infant. Within the literature on infant gaze-following or point-following, a series of carefully controlled studies (Butterworth and Jarrett, 1991; Deák et al., 2000) have shown that ‘behind’ locations are consistently harder to locate than those in front or to the sides. In relation to gaze this effect is apparent in the first half of the second year (Butterworth and Cochran, 1980; Butterworth and Jarrett, 1991). It is possible that the effect may be present even earlier in simpler localization tasks such as locating sounds in space. If this were the case it would constitute not only an early difficulty with space where there was no meeting of the perceptual systems of infant and other (as suggested by Butterworth and Jarrett, 1991) but more simply with space not currently available to the infant’s perception. Although following gaze or pointing to a distal target is clear evidence of triadic joint attention, usually believed to occur after 9 or 10 months of age, some recent studies have argued that forms of joint attention may be evident even in the first 6 or 7 months (Striano and Bertin, 2005; Parise et al., 2010; Rossmanith et al., 2014). Although there have been some anecdotal claims (e.g., Butterworth, 1998) that localizing sound in any direction is possible from very early, there is no data to date about infant ability before 6 months of age, to locate sounds from ‘behind.’ Studies which have explored infant ability to turn ‘behind’ to locate a sound have focused on infants after 6 months (Nadig et al., 2007; Van der Meer et al., 2008) and studies looking at younger ages have explored lateral sounds only, placing the sound right next to the ears (Wertheimer, 1961), 3–4 feet away on either side (Aronson and Rosenbloom, 1971; McGurk and Lewis, 1974), or 20 cm away on either side (Field et al., 1980).

Within the neo-Piagetian tradition of object permanence studies, the perseverative errors in the A not B task are, in a paradoxical way, evidence of infant knowledge and expectation of the location of the specific object. In further development of this methodology using eye-tracking, anticipatory looking before the hidden object reappears was confirmed at age of 10–12 months (Watanabe et al., 2012), 18 months (Forssman et al., 2014) and 12 and 24 months (Johansson et al., 2015). Even in the face of recent perceptual information to the contrary, infants expect that objects or persons remain in the last known location. Auditory localization tasks present an easier challenge to infants than does the A not B task and it is possible that perseverative errors indicating expectation of a specific source of sound may be apparent even earlier.

The present study attempted to construct a semi-naturalistic situation where three groups of infants aged 4, 5 and 7 months, seated on their mothers’ laps, were engaged with one experimenter in front of them, and were then called by a second experimenter’s voice or by the sound of castanets from side and ‘behind’ directions. We predicted that there would be a significant difference between the 4 month group and the 7 month group in the accurate localization of both voice and sound at all directions, as seen in an increase in the percentage of infants successfully ‘finding’ the source of the sound, as well as in the number of repetitions of the sound needed for successful localization. We predicted that at all ages successful localization at the left and right side locations would be greater than at the ‘behind’ location. We predicted, that at the earlier ages, localization of the voice would be superior to the localization of the castanet sound. Lastly, we asked two open-ended questions: one, about the possible occurrence of spontaneous attention shifts between the two experimenters and two, about the possibility of perseverative errors – i.e., looking to the last location – at all ages.

## Materials and Methods

### Participants

Thirty five infants (fifteen 4 month-olds, *M* = 17 weeks 3 days, range 15 weeks 4 days – 19 weeks 0 days; twelve 5 month-olds, *M* = 21 weeks 5 days, range 20 weeks 3 days – 24 weeks 2 days; eight 7 month-olds, *M* = 31 weeks 0 days, range 28 weeks 5 days – 33 weeks 1 day) were recruited from the babies who attended a regular health check at Tamamura Health Centre in Gunma, Japan. The health center covered the population of the whole of Tamamura town.

This study fully complied with the ethical principles of the 1964 Declaration of Helsinki and received ethics approval from the Review Committee of the Gunma Prefectural Women’s University. Flyers about the study were distributed to all who attended the health center. If they expressed interest in the study, they were given full details of the study and were then asked if they wished to participate, and to give oral informed consent in the presence of two researchers. It was customary practice in Gunma at the time this study was conducted (2003) to obtain oral rather than written consent for research. The parents were offered the chance to learn about the results of the study if they wished.

### Setting

**FIGURE 1:**
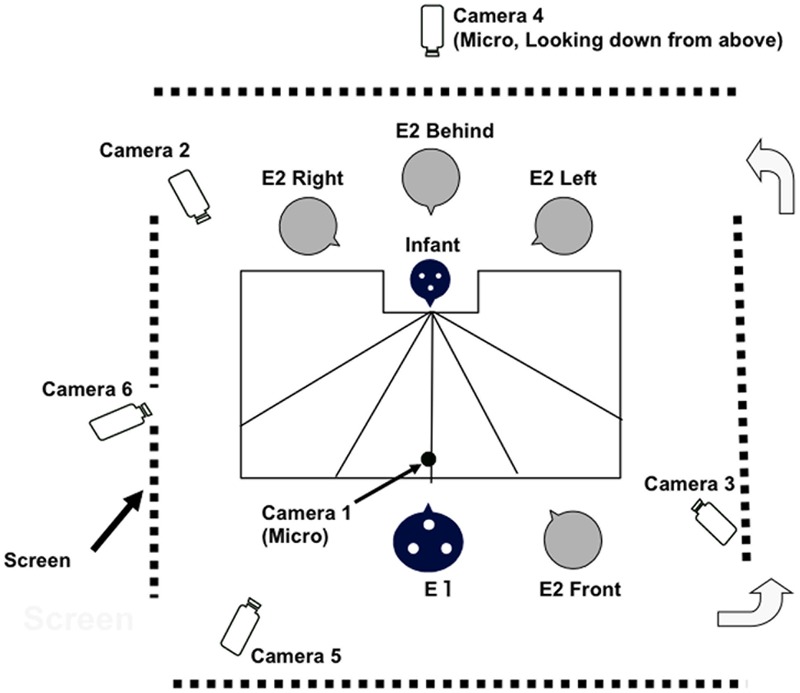
Bird’s – eye view of the experimental setting. The table (60 cm × 120 cm) was cut out to make a space (15 cm × 30 cm) for the infants to fit in.

Infants were observed in a partitioned-off area of the health center. Infants’ behavior and experimenters’ behavior were video-recorded with six video cameras using a 1/30 s time signature (see **Figure 1** for layout). The first camera focused on the infant’s face. The second focused on the face of the first experimenter (E1), the third shot from the infant’s left side, and the fourth took a bird’s-eye view of the whole scene. The pictures taken by these four cameras were integrated into a single videotape recorder (by a Four Video Separate Unit). The other two cameras, focused on the infant’s right side and on the infant’s diagonal front, were used to clarify any ambiguities of response. To assess the direction of the infant’s head and gaze, three yellow markers were placed in a triangle on top of the infant’s head. The bird’s-eye view of the experimenters and the infant, with the lines marked on the table, made it possible to capture the infant’s head movements accurately.

### Procedure

The experimental procedure was similar to the ‘2+1’ situation in the Lausanne trilogue play (Fivaz-Depeursinge et al., 2010), used to study infants’ early triadic interactions with their parents. The ‘2+1’ situation was one of four scenarios used in the Lausanne trilogue play and involves one parent actively engaging with the infant while the other is ‘just present’ taking a ‘third party role.’ In the present study we did not use parents, but two adult females (strangers to the infant). The literature suggests that infants at 7- to 8 months can discriminate between different talkers if the language is the native tongue (Johnson et al., 2011), but that they find it extremely difficult (between 6 and 8 months) to separate the simultaneous speech of two unfamiliar women unless one of the voices is highly familiar to them (Barker and Newman, 2006). Therefore, the procedure in this study was set up to avoid the simultaneous speech of the two strangers, and also to notify to the infants at the start (by presenting Experimenter 2 at the Front position in **Figure 1**) that there were two persons involved.

The infants were seated on their mothers’ laps across the table from E1. After they were seated E1 asked the infant’s name, asked her permission and then placed the marker on the infant’s head. E1 then engaged in face-to-face interaction with the infant. The mothers were instructed to refrain from interacting with the infants or helping them during the experiment. The second experimenter (E2) appeared at the front next to E1 (the Front position), called the infant by name and confirmed eye-to-eye contact, greeted the infant saying hello, waved and then said good-bye. Then E2 ‘disappeared’ from the infant’s sight, moving behind the screen to the Left, Right, or Behind positions as shown in **Figure 1**. E2 called the infant by name outside the infant’s view: squatting down on the infant’s right side and the left side, or standing behind the infant (and the mother, with her shoulders and head higher than the mother’s head so that she could see the infant at all times). The order of the locations Left, Right, and Behind (but not the Front position) was randomized. Each infant was called a maximum number of three times (one trial) at about 2 s intervals from each (Left, Right, and Behind) position. If they successfully found and looked at E2, E2 responded saying, “You found me!” and did not repeat the call from that location. When the sound came from behind, if the infant turned her head back clearly past the Left or Right locations E2 moved her face slightly to make herself more visible. While E2 was calling the infant’s name, E1 was ‘just present.’ After E2 stopped calling, E1 tried to re-engage the infant by saying, “Who called you? I was not the person who called you,” or if they had successfully found E2, E1 said “You found her!”. This procedure was conducted in the exact same pattern, with the castanet sound (a double click repeated up to three times). The order of presentation of voice and castanets was fixed, with voice always occurring first in order to start the experiment with an ‘easier’ and more ‘motivating’ sound.

### Coding and Reliability

#### Localizing Responses

Each infant’s final response within a maximum of three calls/sounds (one trial) from each location (Left, Right, and Behind) was classified into one of three categories from the video tapes: ‘Found’ for turning their head to search and successfully finding E2 or the castanet in E2’s hand, ‘Searched’ for turning their head to search but not finding, and ‘No shift’ for no attention shift away from E1’s face, or for looking down (no turning).

#### Number of Repetitions of Call/Sound

The number of repetitions of the sound (voice and castanet) needed for localization on each successful trial was counted from the videos. The maximum number of repetitions was three for each trial.

#### Last Location Error

An unsuccessful search was coded as a last location error if, on hearing the voice or castanets, infants turned their gaze to the place where E2 had just previously been. On only four occasions in the entire study the infant first turned to the last location but then quickly turned to the correct location. These were not counted as last location errors.

#### Spontaneous Attention Shift from E2 to E1

After finding E2, infants sometimes shifted their gaze to E1 before E1 spoke to them with the words “You found her!”. This spontaneous attention shift was coded for every location from which the voice or sound was presented (Left, Right, Behind, and including Front in this case).

#### Positive Affect

During the ‘spontaneous attention shift from E2 to E,’ the following expressions were coded as demonstrating positive affect: smiles, laughs, signs of happiness or excitement as shown by shaking the body or hitting the table with a smile.

#### Reliability

Agreement of the two coders (Cohen’s kappa), calculated from approximately 20% of the videos (*N* = 6: two infants at each age group) were as follows: κ = 0.95 for the three localization response categories (Found, Searched, No shift), κ = 0.95 for the number of repetitions of the sound, κ = 0.80 for the last location error, κ = 1.00 for the spontaneous attention shift, κ = 1.00 for the positive affect.

## Results

Given the small sample size, non-parametric tests were used for analysis. Fischer’s exact tests were used for comparison of age group differences in localization responses and spontaneous attention shifts, Cochran’s Q test was used for comparison of Left, Right, and Behind position, and McNemar’s test was used for comparison of Voice and Castanet Sound.

### Age, Successful Localization, and Number of Repetitions

Fisher’s exact tests showed significant associations between age and the three localization responses for each location and for both sound types. Multiple comparisons using a Bonferroni correction adjusted alpha level of 0.017 (0.05/3) revealed significant differences between each age group (see **Table 1**). In sum, there was a clear increase in Found responses with age, with significant differences between 4 and 7 months at each location and with each sound type. Significant differences between 4 and 5 months were only found at the Right for Voice and between 5 and 7 months at Behind for both Voice and Sound.

**Table 1:** Frequency of localizing responses at each age for Voice and Castanets from each direction (%).

		Voice			Castanets		
Location		Found	Searched	No shift	Found	Searched	No shift
Left side	4 m (*n* = 15)	5 (33.33)	5 (33.33)	5 (33.33)	4 (26.66)	2 (13.33)	9 (60.00)
	5 m (*n* = 12)	8 (66.67)	4 (33.33)	0 (0.00)	7 (58.33)	1 (8.33)	4 (33.33)
	7 m (*n* = 8)	8 (100.00)	0 (0.00)	0 (0.00)	8 (100.00)	0 (0.00)	0 (0.00)
		Fisher’s exact test: *p* = 0.0089^a^			Fisher’s exact test: *p* = 0.0094^d^		
Right side	4 m (*n* = 15)	4 (26.67)	6 (40.00)	5 (33.33)	6 (40.00)	4 (26.67)	5 (33.33)
	5 m (*n* = 12)	11 (91.67)	0 (0.00)	1 (8.33)	10 (83.33)	1 (8.33)	1 (8.33)
	7 m (*n* = 8)	8 (100.00)	0 (0.00)	0 (0.00)	8 (100.00)	0 (0.00)	0 (0.00)
		Fisher’s exact test: *p* = 0.0003^b^			Fisher’s exact test: *p* = 0.0338^e^		
Behind	4 m (*n* = 15)	0 (0.00)	3 (20.00)	12 (80.00)	0 (0.00)	6 (40.00)	9 (60.00)
	5 m (*n* = 12)	0 (0.00)	5 (41.67)	7 (58.33)	0 (0.00)	5 (41.67)	7 (58.33)
	7 m (*n* = 8)	5 (62.5)	3 (37.5)	0 (0.00)	2 (25.00)	6 (75.00)	0 (0.00)
		Fisher’s exact test: *p* = 0.0001*^c^*			Fisher’s exact test: *p* = 0.0092^f^		

*Multiple comparisons were made using a Bonferroni correction adjusted alpha level of 0.017 (0.05/3). ^a^Between 4 – 7 m (*p* = 0.0087); ^b^between 4 – 7 m (*p* = 0.0032), between 4 – 5 m (*p* = 0.0020); ^c^between 4 – 7 m (*p* = 0.0001), between 5 – 7 m (*p* = 0.0016); ^d^between 4 – 7 m (*p* = 0.0022); ^e^between 4 – 7 m (*p* = 0.0218); ^f^between 4 – 7 m (*p* = 0.0041), between 5 – 7 m (*p* = 0.0112).*

In the 4 month group, although only about one third succeeded in locating E2 or the castanets when the voice or sound came from the Left or the Right, two-thirds of the infants did attempt to search (see **Table 1**). The attempt to search suggests both an interest in E2’s voice and the awareness that the voice did not come from E1. One-third of the 4-month-olds did not make any attempt to search for the voice, in contrast to only 1 of the 5-month-olds and none of the 7-month-olds.

**Table 2** shows the frequency of successful localization (‘Found’) with one call/sound at each age. The frequency of successful localization on the first call or sound increased with age but with a clear bias toward the Left position. There was a significant association between Age and one-call localizing from the Left for the Voice (Fisher’s exact test, *p* = 0.0003) as well as for the Castanets (Fisher’s exact test, *p* = 0.0052). For the Right position there was a significant association with Age for the Voice (Fisher’s exact test, *p* = 0.0348) but not for the Castanets (Fisher’s exact test, *p* = 0.228, ns). The frequency of correct localization was low for the Behind position even in the oldest age group (with no instances of one call localization).

**Table 2:** Frequency of successful localisation with only one call/sound at each age.

	Voice				Castanets			
	4 m	5 m	7 m	Fisher’s exact test	4 m	5 m	7 m	Fisher’s exact test
	*n* = 15	*n =* 12	*n* = 8	*p*	*n =* 15	*n =* 12	*n* = 8	*p*
Left	1	4	7	0.0003	0	1	4	0.0052
Right	1	5	4	0.0348	0	0	1	0.2286
Behind	0	0	0	–	0	0	0	–

### Left and Right Positions versus Behind

Cochran’s *Q* test was used to test whether location (Left, Right, and Behind)-related differences in performance in each age group were significant. Localization categories were collapsed into a dichotomous variable consisting of ‘Found’ versus ‘Others’ (‘Searched’ and ‘No shift’). For the Voice, a significant difference was found in the 5-month-olds with more Found responses to Left and Right than to Behind (*Q* = 16.17, *df* = 2, *p* < 0.05). Only marginal significances (in the same direction) were found in the 4-month-olds (*Q* = 5.25, *df* = 2, *p* = 0.072) and the 7-month-olds (*Q* = 16.17, *df* = 2, *p* = 0.05). On the other hand, in the case of the castanet sound, significant differences were found in all age groups with more Found responses to Left and Right than to Behind (4-month-olds: *Q* = 6.22, *df* = 2, *p* < 0.05, 5-month-olds: *Q* = 14.36, *df* = 2, *p* < 0.01, 7-month-olds: *Q* = 12.00, *df* = 2, *p* < 0.01).

At all ages responses to the ‘behind’ direction tended to be poorer than at the left or right sides. Particularly in the 5-month-olds, that tendency was significant for both sound types. The 5-month-olds efficiently located voice and sound, for all except the ‘behind’ direction. The 7-month-olds demonstrated clearer localization of sounds in all directions and the 4-month-olds poorer localization in all directions.

### Last Location Errors

**FIGURE 2:**
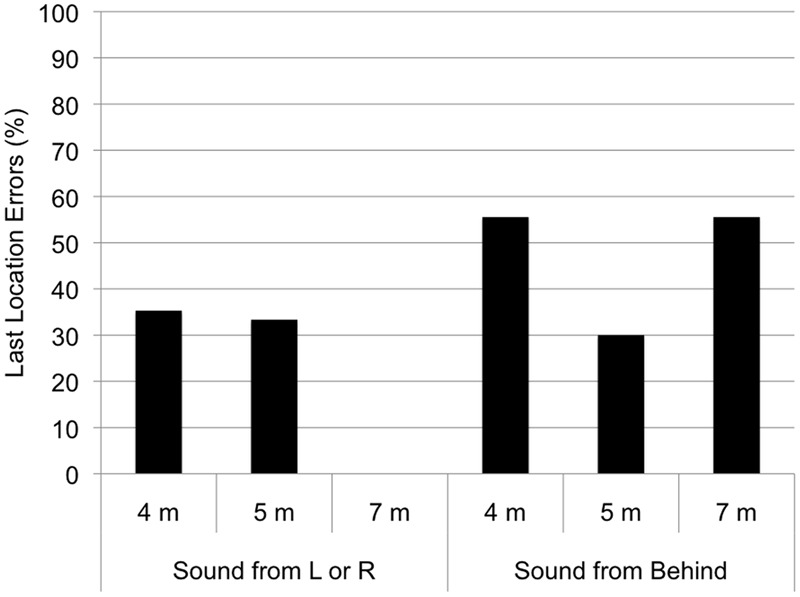
Last location errors at each age for each direction, as a percentage of unsuccessful search attempts.

**FIGURE 3:**
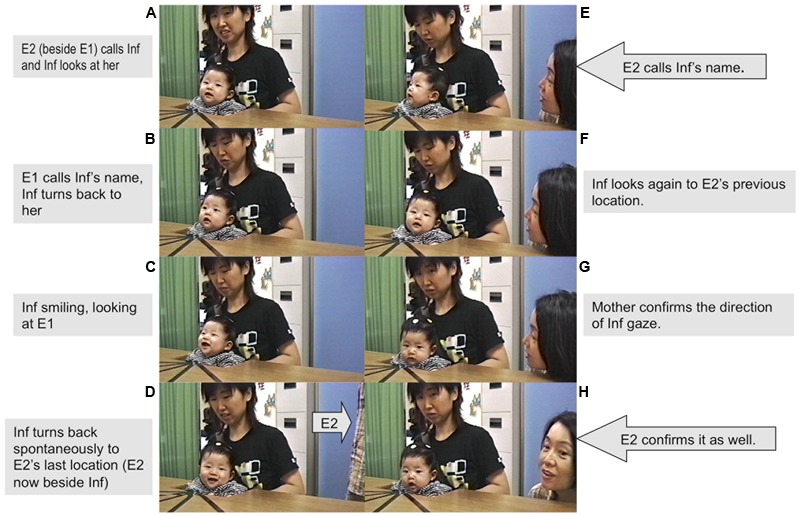
Example of last location error: boy, 16 weeks, searches at previous E2 location. **(A)** Inf (boy), 16 weeks, looking at E2 who had called him from the front (beside E1). **(B)** E1 calls Inf’s name and Inf turns back to her. **(C)** Inf smiles at E1. **(D)** Inf spontaneously turns back to E2’s last location. But at that time, E2 had already moved away, standing beside Inf, and he could not find her. **(E)** Inf turns back to E1; E2 calls Inf’s name. **(F)** Inf looks again at E2’s previous location beside E1. **(G,H)** M and E2 confirm that Inf looking at E2 previous location.

Last location errors during unsuccessful searches occurred at all ages; 61.9% of these occurred in response to the voice, and 38.1% to the sound. In response to Left and Right locations unsuccessful searches only occurred at in the two younger age groups, and in both groups one-third of the unsuccessful searches involved looking at the last location of the voice or sound (35% at 4 months and 33% at 5 months, see **Figure 2**). In the 4 month group, however, these errors primarily occurred when E2 disappeared from the front of the infant and then called from the other sides or back (see **Figure 3**); this was not the case in the 5 month group. In response to Behind locations, all age groups showed some unsuccessful search attempts, and in all age groups, including the 7 month group about half of these attempts involved looking at the last location of the voice or sound (56% at 4 months, 30% at 5 months, and 56% at 7 months).

**Figure 3** shows a sequence of pictures of a gaze-shift to the previous location (front) by a 4 month-old boy (16 weeks, 5 days) when he was called from the left immediately after having been greeted from the front.

### Voice versus Castanet Sound

We used McNemar’s test to explore differences between Voice and Castanet sounds. There were no significant differences at any age or at any location between localization of Voice and Castanets.

### Spontaneous Attention Shifts and Positive Affect

**FIGURE 4:**
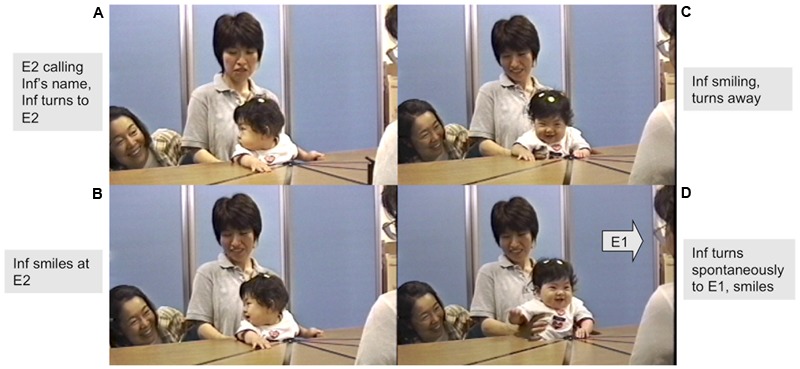
Beginnings of triadic attentional engagement: girl, 29 weeks spontaneously shifts attention with positive affect back to E1 after seeing E2. Inf (girl) 29 weeks, 5 days. **(A)** When called by E2 from her right, she turns to her. **(B)** Inf smiles at E2. **(C,D)** Inf spontaneously turns back to E1 with positive affect smiling, vocalizing and patting the table (before E1 speaks to her).

After finding E2, some infants returned their gaze to E1 even before E1 spoke to them with the words “You found her!” These spontaneous attention shifts to the first partner were especially frequent in the 7-month-olds (in 75% of infants at 7 months as opposed to 13% at 4 months and 8% at 5 months) showing a significant association with age (Fisher’s exact test, *p* = 0.0021). Of these spontaneous attention shifts from E2 to E1, 78.6% occurred in response to the voice and 21.4% to the sound, and 71.4% occurred at the Front position and 28.6% at the Left or Right position. The majority (64%) of the 7 month-olds’ gaze shifts involved multiple shifts – i.e., from E2 **→** E1 **→** E2 and often again to E1. A small proportion (28.6%) of the spontaneous attention shifts at 7 months were accompanied by positive affect. **Figure 4** shows a sequence of stills illustrating the spontaneous gaze shifts of one infant aged 7 months (29 weeks and 5 days).

## Discussion

The present study investigated auditory localization from lateral as well as ‘behind’ locations in a multi-person environment between 4 and 7 months of age. The findings supported two of our hypotheses (regarding increase in success and decrease in need for repetitive calls with age and regarding an advantage for lateral over ‘behind’ directions). However, they failed to support one hypothesis (regarding a Voice over Sound advantage). The presence of last location errors and spontaneous attention shifts suggest, additionally, that localization of sounds may have parallels with perseverative errors in other tasks (such as the A not B task) and may elicit the initiation by infants of simple forms of triadic attentional engagements.

Our first hypothesis regarding age effects was supported. As we predicted, we found significant increases with age in the successful localization of both voice and sound from all directions. Unlike the possibly reflexive neonatal orientation to lateral sounds reported in the literature, the attempts to localize the voice or castanet sounds in this study were much more complex. Nonetheless, even the 4-month-olds turned their heads and attempted to search for the sound, one third of them succeeding when the sound source was from the lateral directions. Even their failure to search when E2’s voice or the sound was heard – i.e., by continuing to look at E1 often with a concentrated look and stilled body suggests that they may have been trying to determine the source of the sound. Their search, particularly their last location errors implied an expectation of the person who had been speaking or making the castanet sound rather than a reflexive response to sound. Seven month-olds accurately and quickly located voice and sound outside of their immediate view, sometimes even from behind. At 7 months their expectation about the origins of the sound was clear; one infant even tracked E2 disappearing into the screen, and then turned back to the direction anticipated from E2’s trajectory and waited for her to reappear from behind the screen. The age effects in this study were even clearer when considering success at localizing after one call: the increase between 4 and 7 months was clearly linear at least for localizing Voice. However, there was a clear bias toward the Left position. This could have been created by E2 appearing to the left of E1 and disappearing toward the Left.

These improvements with age, that is, both the increase in accuracy of localization and the decrease in number of repetitions between 4 and 7 months provides partial support for previous findings of an increase in localization ability after 4 months (Muir et al., 1979, 1989; Field et al., 1980). The complexity of the experimental situation and of the infant responses in the present study suggests that at least after 4 months infant responses to sound may indeed be cortically mediated as suggested by Muir and Clifton (1985). The increasingly efficient localization of the sources of voice and sound in the 5 and 7 month-olds is also consistent with findings from studies using functional near-infrared spectroscopy (fNIRS) showing voice-sensitivity in the temporal cortex at 4–7 months of age (Grossmann et al., 2010; Grossman and Friederici, 2012; Lloyd-Fox et al., 2012).

A number of studies of inter-modal perception in early infancy have provided evidence that infants are sensitive to the temporal synchrony of sound and vision (Lewkowicz, 1986; Bahrick, 1988), and to the discrepancy between phonetic information in lips and in voice (Kuhl and Meltzoff, 1984; Patterson and Werker, 1999). However, they are not so sensitive to spatial discrepancy (McGurk and Lewis, 1974; Condry et al., 1977). When being called from outside of their immediate visual field, the 4-month-olds in this study when they did not search, kept looking intently at E1 in front of them, as if asking E1 “Are you calling me?” despite E1’s silent ‘third party role.’ Their responses seemed to be dominated by their visual engagement with E1. Even when the 4-month-olds noticed that the voice was coming from another source and did attempt to search, their unsuccessful searches often resulted in looking in the previous location where they had seen E2 calling or making the sound. This kind of visual dominance may have some connection to the ‘ventriloquism effect,’ the well-known illusion in adult perception characterized as a mis-localization of auditory events, due to being captured by a visual signal. This effect is described as a spatial discrepancy between visual and auditory signals which can be related to a single source, event, or object (Radeau and Bertelson, 1974). Bedford (2001) pointed out that an object identity decision is essential for producing the ventriloquism effect. Young infants’ high tolerance to audio-visual spatial discrepancy, presented in the ‘split mother’ experiments (McGurk and Lewis, 1974; Condry et al., 1977) may be explained by this illusion. However, in this study, since E2 first appeared next to E1 in front of the infants and then disappeared from their view for calling from other locations, the infants could easily recognize that there were two strangers (E1 and E2) involved. The visual dominance of the 4-month-olds revealed in the present study is therefore not the ventriloquism effect but may be a feature of a less mature developmental stage before successful locating of voice and sound is achieved.

In keeping with the finding that non-visible or non-shared space is challenging, with gaze following to ‘behind’ locations occurring only after 18 months (Butterworth and Jarrett, 1991), we also found that localization of sounds emerging from behind the infant was more difficult than of sounds emerging from the sides at all ages. Butterworth (1998) noted that ‘By 18 months, although babies do not search behind them when there are targets in the field of view, they will do so if the visual field is empty.’ However, in the present study infants were already showing a partial awareness of space behind them by 7 months, despite an engaging visual target (E1) in the field of view. It is possible that the younger ages were limited by less postural control of the upper body (given the suggestion that rotation skills may be necessary for 6–9 month-olds to respond to their mothers’ calls from behind, Van der Meer et al., 2008). Nonetheless, the presence of some successful ‘behind’ localization at 7 months despite the attractive alternative target available to the infant, suggests that the integration of the ‘visual space’ in front and the ‘auditory space’ behind may be achieved much earlier than Butterworth (1998) has suggested. The use of calling the infant’s name and the fact that the ‘stimuli’ in the present study were actually a person’s voice or a person’s active sound production may have enabled this sensitivity to space behind, earlier than in previous studies where the stimulus to be searched for was not produced by a person. It is very likely that although grasping space ‘behind’ is more difficult than space more easily available perceptually, the difficulty is task specific. In other words, the emergence of gaze following to ‘behind’ locations not before 18 months, may be due to the difficulty of gaze following rather than to the difficulty with the grasp of space.

Contrary to our prediction, we did not find clear support for our hypothesis that localizing voice would be easier than localizing the castanet sounds. The castanet sounds may have acted as a social stimulus for the infants, not very different from the calling, because they were clearly made by E2. In fact some infants in successful trials turned, first looked at E2 who made the sounds, and then looked down at the castanets in her hands and some infants did the reverse – first looking at the castanets and then at E2’s face. In other words, the infants may have expected to see a person, even when hearing the castanet sounds. It is also possible that in auditory localization tasks there is no Person advantage; the earlier findings of Person permanence emerging earlier than Object permanence (Bell, 1970; Bowlby, 1980/1998) have been challenged (Jackson et al., 1978) and are at best contradictory. However in order to distinguish interest in voices from interest in other sounds, a condition in which a castanet sound is played absent of an experimenter might have been an important control condition. The lack of difference in infant response to Voice and Sound raises this important question: would infants respond to sounds produced by persons in a different way than to sounds evidently produced by machines (such as a moving toy for instance)? Future research is needed to clarify this question.

The occurrence of last location errors was rather higher in the 4-month-olds than in the 5-month-olds and not present at all in the 7-month- olds for the lateral directions. Although the classical A-not-B task requires infants’ reaching skills, a looking version of the task have been developed and showed comparable performance by 8–9 months of age (Bell and Adams, 1999; Cuevas and Bell, 2010). Similarly, our results suggest that such perseverative errors are not attributable to the type of measure (reaching or looking), but to the expectation of the person or object, and may also be task specific. That is, in simple localization tasks, the perseveration may be present much earlier than in more cognitively challenging tasks such as object permanence. This conclusion is crucially supported by our finding that for the behind location (which was also difficult for the 7-month-olds) all the groups showed these errors. Further research is needed to confirm this interpretation with a larger sample.

Lastly, the rapid gaze shifts with positive affect from E2 to E1 seen in some infants particularly at 7 months, after they found E2 and before E1 called them back, are strongly suggestive of an early ability to initiate triadic interaction (Mundy et al., 2003) with two adult strangers. A typical triadic interaction in joint attention can often be described as a POP (Person-Object-Person) interaction, considered to appear around 9 or 10 months. However, in the case of PPP (or Person-Person-Person) interactions where the other is looking at another person rather than an object, even babies at 3–4 months have been shown to follow gaze (Tremblay and Rovira, 2007). The Lausanne trilogue study (Fivaz-Depeursinge et al., 2010) using a PPP situation showed 3- to 4-month-olds’ capacity to simultaneously communicate with two partners (father and mother) through rapid multiple gaze transitions (5 or 3 s) between parents during interactions (McHale et al., 2008). In the present study we found these clear multiple alternations at 7 months, with very occasional incidence before that age. Together these findings provide a challenge to the widespread assumption that triadic attentional engagements – and in particular the initiation of typical joint attention – is only possible after 9–10 months of age.

There could be another explanation: the infants may have been simply shifting their attention from one adult to another. Even if this were so, this stimulus-driven shifting might become the first step to involvement in the interaction with the two adults. The first experimenter (E1) always pointed out the connection to the infant by saying, “You found her,” reacting to their response as if they were indeed involved in triadic relations. The adult’s reactions may have a scaffolding effect on their subsequent development. Nonetheless, several studies report that understanding triadic relations is not an all-or-none achievement at the end of the first year; it is present in various forms from 5 to 7 months (Striano and Bertin, 2005) and even earlier (Parise et al., 2010; Rossmanith et al., 2014) and the present findings lend support to the conclusion from those studies that the cognitive underpinnings of triadic attentional engagements are available already at 7 months of age. Future research could use the present localization task with a larger sample and with an additional age group of 9–10 month-olds where we would expect more frequent spontaneous attention shifts between E1 and E2. The advanced motor skills of the 7-month-olds may also contribute to these shifts in the present study, enabling quicker turning and more rapid interactions with more than one person. In typical home environments there is not only greater chaos than in a controlled lab environment but also multiple persons and targets of interest. The present study suggests the importance of using multi-person and complex environments studying even simple cognitive skills.

## Author Contributions

The experiment was designed and conducted by EK and SA. The data was analyzed and interpreted by EK, SA, and VR. The article was written by EK and VR.

## Conflict of Interest Statement

The authors declare that the research was conducted in the absence of any commercial or financial relationships that could be construed as a potential conflict of interest.
